# St. George's Hospital

**Published:** 1903-01-03

**Authors:** 


### HOSPITAL ADMINISTRATION.

### CONSTRUCTION AND ECONOMICS.

### ST. GEORGE'S HOSPITAL.

#### Wanted a Committee of Management.

We had the pleasure last week of going over St. George's Hospital and inspecting most of the departments. St. George's is a hospital which has been criticised on the ground that it had fallen behind in regard to its equipment, appliances and furniture, and the accommodation which it provides in the casualty department and elsewhere. We were anxious to ascertain what was being done by the Governors, and to what extent improvements had to be effected. We had the advantage of the presence of Mr. A. William West, one of the treasurers, whom we were glad to welcome as a young and active worker in the hospital field, and of Miss Florence Smedley, the matron, who has done much good work since she assumed office, and who brings a clear and intelligent mind to bear upon the organisation and books under her control. Everywhere there was evidence that tentatively, but surely, St. George's Hospital is being modernised and brought up to date so far as it is possible to accomplish these ends in a building erected, as to the greater portion, before hospital construction was understood as it is to day. St. George's is about the last of the big hospitals to continue the open board system of government, and we cannot help feeling that the time has arrived when it is desirable that this system should be changed in favour of a Committee of Management, which will be charged with the responsibility and duty of administering the affairs of the hospital and reporting to the whole body of Governors once a quarter. The truth is that the more active spirits who are interested in hospitals will not devote themselves continuously to the work unless they are vested with adequate responsibility. That responsibility can only be secured by entrusting the management of a hospital to a committee elected by and reporting to the Governors. If St. George's was to have such a committee it would attract to the institution a number of young and active men. All the additional income it requires would speedily be forthcoming, and so the whole institution would greatly increase in prosperity and efficiency. We will first deal with the strong points connected with St. George's Hospital, and then with the weak ones.

#### The New Equipment.

The work of renewing the furniture and equipment of the hospital is proceeding rapidly, and on the whole successfully. The bedsteads are of modern pattern, and although we think it would have been better to have spent about £6 a bed, so as to have had the Guy's Hospital pattern, which is probably the best form of bedstead to be found in any hospital, still the cheaper form introduced at St. George's, at about half the cost, is a great improvement on the old ones, and the fact that they are not more than 2 feet 9 inches wide is a good feature, seeing that that width is ample for the comfort of the patients, that it tends to improve the atmospheric conditions of the ward by allowing freer circulation of air, and additional clear superficial and wall space between the beds. The narrower beds have, however, one disadvantage, and we observed that that disadvantage was present in some of the wards of St. George's Hospital, a fact which we hope will be speedily altered. The narrower beds will fit into corners more readily than the wider ones, but it is quite unjustifiable to have beds pushed up close to the end and side walls of a ward, as is the case at present in the Princess and other wards of St. George's Hospital, without any window space intervening. From the earliest days those who have closely followed the hygienic conditions of a hospital ward know, that beds so situated are necessarily unhealthy, and that they ought never to be occupied by a patient with an open wound. Indeed, we are of opinion, that they should never find a place at all in a modern hospital ward conducted with due regard to the highest efficiency. At St. George's Hospital the typhoid cases are treated in the general medical wards, but each typhoid case has a red collar to his shirt, and everything which is used by the typhoid patient is kept in a separate glass cupboard at the end of his bed, so that there shall be no danger of infection to anybody. Thus, each typhoid patient has a separate medicine glass, separate feeding mug, thermometer, dishes, plates, cups, and everything used for food, with the usual ward crockery required by a patient who is dangerously ill and confined to bed. Each of these requisites is of a special pattern and colour, distinct from those used by the other patients. Another good point is the feature made in each of the wards of the testing rooms, in some cases by the utilisation of a window space with closing doors, in which all that relates to the testing can be carried on with the necessary precautions and despatch. All basins and jugs have been abolished from the wards and a sink with special taps for hot and cold water and other excellent fittings have been substituted, which we think on the whole may be regarded as a marked improvement. The new teak lockers are made of an approved pattern, and also serve as bed tables. In lieu of the old deal benches which were kept under the patients' beds and entailed a considerable expense from the amount of scrubbing they involved, teak stools are being substituted. The new chairs in the wards are attractive in appearance and very comfortable. Electric light has been supplied to each of the wards and is so arranged as to enable the patients to read comfortably in bed. Each bed has its own special plug, to which a larger lamp can be readily fixed, whereby the strongest light can be brought to bear upon a patient requiring to be dressed or when a careful examination has to be made.

#### The New Kitchen and Linen Rooms.

This kitchen, which is at the top of the hospital, is in many respects a model hospital kitchen. Electricity has been pressed into the service, and the whole plan of arrangement, not only in the kitchen, but in the pastry rooms, sculleries, vegetable larder and hot-plates with their service sections are well worthy of inspection by anyone interested in this branch of hospital economy. St. George's always labours under great disadvantages in regard to space, but the meat larder should be ventilated from the roof, as at present it is very deficient in this respect. The linen-room and the adjacent store-room for medical dressings, etc., are also very good. They show considerable intelligence in their arrangements, are admirably kept, and reflect great credit upon everybody connected with them. We are told, and we can well believe, that the system of issue and return is so good that at a recent stock-taking every article in the large stock in use in this hospital exactly balanced. This is surely a triumph for the ladies in whose control these departments are vested.

### The Nurses' Quarters.

There has been a revolution in the accommodation provided for the nurses at St. George's Hospital. It is no doubt a misfortune that a number of the staff have to be housed in a home some distance away from the hospital, but in rearranging the actual accommodation available for the nurses within the hospital itself, everything has been done to make that accommodation as efficient as possible. Instead of having cubicles, every nurse is provided with a separate room upon a plan which shows that where sufficient thought and planning are brought to bear, it is possible in all hospitals to abolish cubicles and substitute separate rooms for them. The nurses' sitting-room is one of the most attractive and comfortable to be found in any hospital, and we believe that the Governors who have had the wisdom to expend the necessary funds upon this department, have their reward in the marked improvement which is everywhere apparent in the *esprit* of the nurses and the type of woman to be met with in each of the wards. One practical point we noticed in regard to the bath of vitreous enamelled steel of the Doulton pattern, which costs some £7, and is, we think, on the whole the most suitable pattern of bath for hospitals. One bath we inspected had been in use from three to four years, and was in as perfect condition as the day when it was first supplied, although it had been in constant use throughout the whole period by 10 nurses at least. Another of the same pattern of bath fixed elsewhere in the nurses' quarters showed signs of wear on the bottom, to which we direct Messrs. Doulton's attention, as it is due entirely to the fixing, the broad end of the bath being a little too low, so that there is generally some water left in the bath after use. There can be no doubt that the bath to a hospital resident is one of the essentials to good health, and the form of bath used should always be made so attractive and pleasant to look upon, that each user will carry away so agreeable an impression of it, that unconsciously, every opportunity to use it frequently will be welcomed. In case anyone may think this is putting the matter too high, we would ask them to take the trouble to go to Tunbridge Wells General Hospital, and visit the baths in that institution, and then ask themselves whether they could, without a strong effort of self-discipline, enter a bath presenting the appearance they do. We can quite believe that at Tunbridge Wells both patients and nurses must often have turned away in disgust, owing to the unattractive appearance of these neglected and filthy-looking bath tubs. In every institution each bath should have a large brush suspended near it, with a request that, after use, everybody will see that the bath is left perfectly clean. This is done at St. George's, in the nurses' quarters, with the excellent results we have had the pleasure to record.

### Weak Points Requiring Immediate Attention.

And here we may say that the old Rufford baths in the wards, which have been greatly knocked about, should either be re-enamelled or, if this cannot be done, that the worst of them should be removed and vitreous enamelled steel baths of the Doulton pattern substituted for them. Where the enamel has perished on the top, a wood moulding might be fixed. Speaking generally, most of the patients' baths in the wards at St. George's Hospital should be renewed, for otherwise visitors will continue to gain the impression that the equipment is faulty. This remark also applies to the bathroom furniture at St. George's Hospital, which requires immediate renewal; some of the chairs are positively filthy looking, and a very small sum would speedily put this matter right. Each of the bathrooms is crowded with mackintoshes and other things, which give to them a disorderly appearance. We are aware, that this is due to the fact that there is no space at present available for the accommodation elsewhere of articles of this kind, but that accommodation can and should be found by a re-arrangement of the space now occupied by linen cupboards and by other means. It will not be easy no doubt to accomplish this, but it is capable of accomplishment, and we have every confidence that Mr. West will speedily find a way.

We would further suggest that the present screens, and especially the old roller towelling which is used to cover them, should be abolished from the wards. They give a workhouse and neglected appearance even to the refurnished wards, which is wholly undesirable and unnecessary. No doubt these screens have been in use for half a century or more, and possibly they may have an attraction for old habitués who will miss them when they visit the wards. Still a stranger, who is accustomed to modern hospital fittings, must view a blemish of this kind with amazement, seeing that the remedy would entail a very small expenditure, and that the whole appearance of every ward is at present spoilt by allowing ugliness to be perpetuated, by the retention of these screens dating from about the time of Noah's Ark. The structural difficulties of St. George's are considerable, but the bad impression which a visitor may gain from the fact that some wards on different levels are connected by archways and steps, could be obviated by the exercise of a little care and artistic skill in regard to decorative effects.

### Casualty Department and Operation Room.

The Surgery of St. George's Hospital is too small, and the fittings are out of date. This same remark applies to the room used for ophthalmic cases and dental purposes. The recovery rooms, too, in connection with the casualty operation room are not sufficiently well ventilated, and this portion of the hospital should be taken in hand without delay and reconstructed. We satisfied ourselves that without in any way interfering with light and air very substantial improvements could be introduced, which would speedily tend to make the surgery and other rooms mentioned up to date, adequate in size and replete with all necessary fittings. In dealing with an old block of buildings like St. George's Hospital, considerable ingenuity and devotion is called for on the part of the architect, who should further be possessed of a precise knowledge of hospital requirements. Given these, there need be neither difficulty, great expense, nor delay in removing the objectionable features of this department, to the great comfort and assistance of the working staff and the well-being of the patients.

The end of the whole matter is, that instead of taking two bites at a cherry the Governors of St. George's Hospital should prepare a scheme, as has been done at the London, embracing every point where structural alteration is called for, or equipment is defective. The cost of carrying out each and all of these matters should be set forth in plain figures, and some of the more wealthy of the Governors might be asked to defray the cost of one or more items, which they might select from the list. We make bold to say, that if this course is adopted, and the changes we have indicated are promptly made, within twelve months St. George's Hospital may be made as efficient and as well equipped as any modern hospital need be. This would indeed be a triumph in view of the difficulties surmounted and the results achieved.

